# Ethyl 2-acetoxy­methyl-1-phenyl­sulfonyl-1*H*-indole-3-carboxyl­ate

**DOI:** 10.1107/S1600536809029985

**Published:** 2009-08-08

**Authors:** B. Gunasekaran, Radhakrishnan Sureshbabu, A. K. Mohanakrishnan, G. Chakkaravarthi, V. Manivannan

**Affiliations:** a Department of Physics, AMET University, Kanathur, Chennai 603 112, India; b Department of Organic Chemistry, University of Madras, Guindy Campus, Chennai 600 025, India; c Department of Physics, CPCL Polytechnic College, Chennai 600 068, India; d Department of Research and Development, PRIST University, Vallam, Thanjavur 613 403, Tamil Nadu, India

## Abstract

In the title compound, C_20_H_19_NO_6_S, the phenyl ring of the phenyl­sulfonyl group makes a dihedral angle of 83.35 (5)° with the indole ring system. The mol­ecular structure exhibits a number of short intramolecular C—H⋯O contacts.

## Related literature

For the biological activity of indole derivatives, see: Andreani *et al.* (2001 ▶); Quetin-Leclercq (1994 ▶); Mukhopadhyay *et al.* (1981 ▶); Singh *et al.* (2000 ▶). For related structures, see: Chakkaravarthi *et al.* (2007 ▶, 2008 ▶); Gunasekaran *et al.* (2009 ▶); For graph-set notation, see: Bernstein *et al.* (1995 ▶).
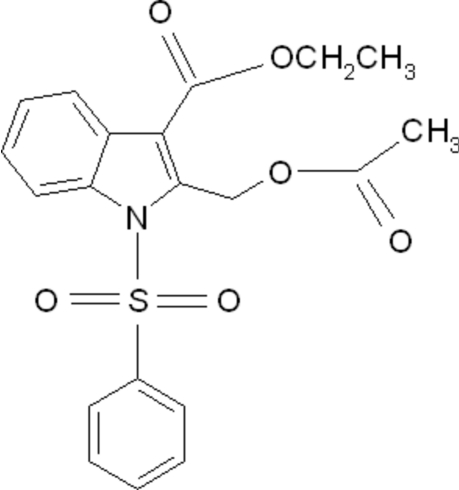


## Experimental

### 

#### Crystal data

C_20_H_19_NO_6_S
*M* *_r_* = 401.42
Orthorhombic, 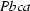

*a* = 18.9097 (6) Å
*b* = 7.9737 (2) Å
*c* = 24.7877 (7) Å
*V* = 3737.50 (18) Å^3^
*Z* = 8
Mo *K*α radiation
μ = 0.21 mm^−1^
*T* = 295 K
0.25 × 0.20 × 0.20 mm

#### Data collection

Bruker APEXII CCD diffractometer
Absorption correction: multi-scan (*SADABS*; Sheldrick, 1996 ▶) *T*_min_ = 0.949, *T*_max_ = 0.959
28247 measured reflections
5788 independent reflections
3533 reflections with *I* > 2σ(*I*)
*R*_int_ = 0.031

#### Refinement

*R*[*F* ^2^ > 2σ(*F* ^2^)] = 0.051
*wR*(*F* ^2^) = 0.153
*S* = 1.01
5788 reflections
255 parameters
H-atom parameters constrained
Δρ_max_ = 0.28 e Å^−3^
Δρ_min_ = −0.35 e Å^−3^

### 

Data collection: *APEX2* (Bruker, 2004 ▶); cell refinement: *SAINT* (Bruker, 2004 ▶); data reduction: *SAINT*; program(s) used to solve structure: *SHELXS97* (Sheldrick, 2008 ▶); program(s) used to refine structure: *SHELXL97* (Sheldrick, 2008 ▶); molecular graphics: *PLATON* (Spek, 2009 ▶); software used to prepare material for publication: *SHELXL97*.

## Supplementary Material

Crystal structure: contains datablocks global, I. DOI: 10.1107/S1600536809029985/gk2226sup1.cif

Structure factors: contains datablocks I. DOI: 10.1107/S1600536809029985/gk2226Isup2.hkl

Additional supplementary materials:  crystallographic information; 3D view; checkCIF report

## Figures and Tables

**Table 1:** Hydrogen-bond geometry (Å, °)

*D*—H⋯*A*	*D*—H	H⋯*A*	*D*⋯*A*	*D*—H⋯*A*
C2—H2⋯O5	0.93	2.57	3.446 (2)	157
C6—H6⋯O2	0.93	2.50	2.875 (3)	105
C8—H8⋯O2	0.93	2.42	2.993 (3)	120
C11—H11⋯O3	0.93	2.48	3.003 (3)	116
C18—H18*A*⋯O4	0.97	2.31	2.886 (3)	117
C18—H18*B*⋯O1	0.97	2.30	2.793 (3)	111

**Fig. 1.**
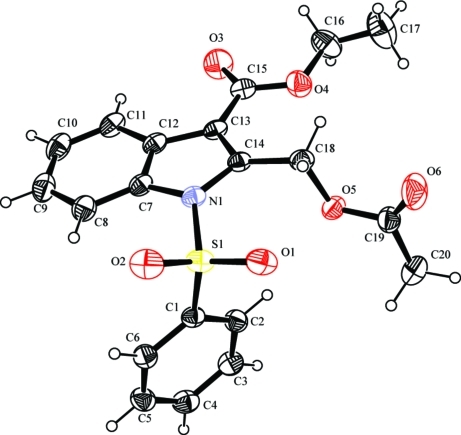
The molecular structure of the title compound with atom labels and 30% probability displacement ellipsoids for non-H atoms.

## supplementary crystallographic information

### Comment

Indole derivatives exhibit antibacterial, antifungal (Singh *et al.*, 2000) and antitumour activities (Andreani *et al.*, 2001). Some of the indole alkaloids extracted from plants possess interesting cytotoxic and antiparasitic properties (Quetin-Leclercq, 1994; Mukhopadhyay *et al.*, 1981).

The geometric parameters of the title compound (Fig. 1) agree well with reported similar structures (Chakkaravarthi *et al.*, 2007, 2008); (Gunasekaran *et al.*, 2009). The phenyl ring makes a dihedral angle of 83.35 (5) ° with the indole ring system. The sum of the bond angles around N1 [356.99 (5)°] indicate the *sp*^2^ hybridized state of atom N1 in the molecule.

A distorted tetrahedral geometry [O1—S1—O2 = 120.74 (10) ° and O1—S1—N1 = 106.73 (8) °] around S1 is observed. The widening of the angles may be due to repulsive interactions between the two short S=O bonds. The torsion angles O1—S1—N1—C14 and O2—S1—N1—C7 [20.02 (18) ° and -51.75 (15) ° respectively] indicate the *syn* conformation of sulfonyl moiety.

The molecular structure is stabilized by weak intramolecular C—H···O interactions. The C6—H6···O2 interaction generate an S(5) graph set motif. The C8—H8···O2, C11—H11···O3, C18—H18A···O4 & C18—H18B···O1 interactions generate S(6) graph set motif and C2—H2···O5 interaction generate an S(8) graph set motif. The C6—H6···O2 and C8—H8···O2 interactions together constitute a pair of bifurcated acceptor bonds generating a ring of graph set *R*_2_^1^(9) (Bernstein *et al.*, 1995).

### Experimental

Ethyl 2-(bromomethyl)-1-(phenylsulfonyl)-1*H*-indole-3-carboxylate (1 g, 2.4 mmol) was dissolved in dry dimethylformamide (10 ml). To this potassium acetate (0.47 g, 4.8 mmol) was added under nitrogen atmosphere. The reaction mixture was allowed to stir for 5 hr at room temperature. Then, the reaction mixture was poured over crushed ice (100 g) containing 1 mL of conc. HCl. The precipitated solid was filtered off and the solid was washed with water (3 *x* 20 ml) and dried. The product was recrystallized from methanol. Yield: 0.7 g (74%), m.p. 361–363K.

### Refinement

H atoms were positioned geometrically and refined using riding model with C—H = 0.93Å and *U*_iso_(H) = 1.2Ueq(C) for aromatic C—H, C—H = 0.97Å and *U*_iso_(H) = 1.2Ueq(C) for CH2, C—H = 0.96Å and *U*_iso_(H) = 1.5Ueq(C) for CH3.

### Crystal data

**Table:**

C_20_H_19_NO_6_S	*F*(000) = 1680
*M**_r_* = 401.42	*D*_x_ = 1.427 Mg m^−^^3^
Orthorhombic, *P**b**c**a*	Mo *K*α radiation, λ = 0.71073 Å
Hall symbol: -P 2ac 2ab	Cell parameters from 6552 reflections
*a* = 18.9097 (6) Å	θ = 2.7–27.1°
*b* = 7.9737 (2) Å	µ = 0.21 mm^−^^1^
*c* = 24.7877 (7) Å	*T* = 295 K
*V* = 3737.50 (18) Å^3^	Block, colourless
*Z* = 8	0.25 × 0.20 × 0.20 mm

### Data collection

**Table:**

Bruker APEXII CCD diffractometer	5788 independent reflections
Radiation source: fine-focus sealed tube	3533 reflections with *I* > 2σ(*I*)
graphite	*R*_int_ = 0.031
Detector resolution: 0 pixels mm^-1^	θ_max_ = 31.6°, θ_min_ = 2.0°
ω and φ scans	*h* = −27→20
Absorption correction: multi-scan (*SADABS*; Sheldrick, 1996)	*k* = −11→8
*T*_min_ = 0.949, *T*_max_ = 0.959	*l* = −36→36
28247 measured reflections	

### Refinement

**Table:**

Refinement on *F*^2^	Primary atom site location: structure-invariant direct methods
Least-squares matrix: full	Secondary atom site location: difference Fourier map
*R*[*F*^2^ > 2σ(*F*^2^)] = 0.051	Hydrogen site location: inferred from neighbouring sites
*wR*(*F*^2^) = 0.153	H-atom parameters constrained
*S* = 1.01	*w* = 1/[σ^2^(*F*_o_^2^) + (0.0697*P*)^2^ + 1.0858*P*] where *P* = (*F*_o_^2^ + 2*F*_c_^2^)/3
5788 reflections	(Δ/σ)_max_ < 0.001
255 parameters	Δρ_max_ = 0.28 e Å^−^^3^
0 restraints	Δρ_min_ = −0.35 e Å^−^^3^

### Fractional atomic coordinates and isotropic or equivalent isotropic displacement parameters (Å^2^)

**Table:**

	*x*	*y*	*z*	*U*_iso_*/*U*_eq_	
C1	0.68111 (9)	0.2922 (2)	0.13401 (7)	0.0409 (4)	
C2	0.66359 (10)	0.3751 (3)	0.18121 (7)	0.0483 (4)	
H2	0.6165	0.3879	0.1913	0.058*	
C3	0.71703 (11)	0.4382 (3)	0.21287 (8)	0.0541 (5)	
H3	0.7062	0.4961	0.2444	0.065*	
C4	0.78647 (11)	0.4159 (3)	0.19792 (9)	0.0558 (5)	
H4	0.8224	0.4567	0.2199	0.067*	
C5	0.80341 (11)	0.3345 (3)	0.15112 (9)	0.0552 (5)	
H5	0.8506	0.3214	0.1413	0.066*	
C6	0.75066 (11)	0.2720 (3)	0.11846 (8)	0.0483 (4)	
H6	0.7617	0.2170	0.0865	0.058*	
C7	0.60056 (10)	0.4924 (2)	0.03978 (6)	0.0428 (4)	
C8	0.66197 (11)	0.4833 (3)	0.00964 (7)	0.0541 (5)	
H8	0.6895	0.3868	0.0088	0.065*	
C9	0.68026 (13)	0.6250 (3)	−0.01919 (8)	0.0637 (6)	
H9	0.7214	0.6239	−0.0397	0.076*	
C10	0.63942 (13)	0.7675 (3)	−0.01839 (8)	0.0625 (6)	
H10	0.6535	0.8601	−0.0385	0.075*	
C11	0.57857 (12)	0.7761 (3)	0.01128 (7)	0.0525 (5)	
H11	0.5511	0.8729	0.0114	0.063*	
C12	0.55875 (10)	0.6358 (2)	0.04138 (6)	0.0430 (4)	
C13	0.49985 (10)	0.6010 (2)	0.07648 (6)	0.0413 (4)	
C14	0.50652 (9)	0.4409 (2)	0.09542 (6)	0.0404 (4)	
C15	0.44401 (11)	0.7252 (3)	0.08755 (7)	0.0494 (5)	
C16	0.33828 (13)	0.7855 (4)	0.13344 (13)	0.0798 (8)	
H16A	0.3227	0.8383	0.1002	0.096*	
H16B	0.3533	0.8727	0.1582	0.096*	
C17	0.28112 (17)	0.6899 (4)	0.15688 (15)	0.1048 (11)	
H17A	0.2638	0.6106	0.1309	0.157*	
H17B	0.2436	0.7644	0.1671	0.157*	
H17C	0.2980	0.6313	0.1882	0.157*	
C18	0.45723 (10)	0.3415 (3)	0.12950 (7)	0.0471 (4)	
H18A	0.4095	0.3849	0.1262	0.057*	
H18B	0.4573	0.2250	0.1182	0.057*	
C19	0.45014 (11)	0.2553 (3)	0.22036 (8)	0.0537 (5)	
C20	0.48239 (14)	0.2728 (4)	0.27501 (9)	0.0738 (7)	
H20A	0.4577	0.2020	0.3001	0.111*	
H20B	0.4789	0.3874	0.2866	0.111*	
H20C	0.5312	0.2403	0.2735	0.111*	
N1	0.56802 (8)	0.37097 (19)	0.07288 (6)	0.0430 (3)	
O1	0.56787 (8)	0.10956 (18)	0.12805 (7)	0.0627 (4)	
O2	0.64517 (9)	0.13016 (19)	0.04850 (6)	0.0663 (4)	
O3	0.44205 (10)	0.8600 (2)	0.06699 (8)	0.0857 (6)	
O4	0.39629 (8)	0.6727 (2)	0.12256 (6)	0.0641 (4)	
O5	0.48159 (7)	0.35577 (17)	0.18482 (5)	0.0502 (3)	
O6	0.40269 (11)	0.1643 (3)	0.20854 (7)	0.0913 (6)	
S1	0.61405 (3)	0.20341 (6)	0.09468 (2)	0.04808 (15)	

### Atomic displacement parameters (Å^2^)

**Table:**

	*U*^11^	*U*^22^	*U*^33^	*U*^12^	*U*^13^	*U*^23^
C1	0.0439 (9)	0.0323 (10)	0.0464 (8)	0.0010 (7)	−0.0041 (7)	0.0052 (7)
C2	0.0451 (10)	0.0481 (12)	0.0516 (9)	0.0023 (9)	0.0002 (8)	0.0016 (8)
C3	0.0603 (12)	0.0513 (13)	0.0508 (10)	−0.0009 (10)	−0.0050 (9)	−0.0036 (9)
C4	0.0541 (12)	0.0504 (13)	0.0631 (11)	−0.0073 (9)	−0.0138 (9)	0.0040 (9)
C5	0.0441 (10)	0.0516 (13)	0.0698 (12)	−0.0025 (9)	0.0003 (9)	0.0104 (10)
C6	0.0504 (10)	0.0405 (12)	0.0541 (9)	0.0027 (8)	0.0030 (8)	0.0026 (8)
C7	0.0524 (10)	0.0400 (11)	0.0359 (7)	−0.0054 (8)	−0.0076 (7)	−0.0011 (7)
C8	0.0604 (12)	0.0565 (13)	0.0454 (9)	−0.0010 (10)	0.0001 (8)	−0.0051 (9)
C9	0.0693 (14)	0.0767 (17)	0.0452 (10)	−0.0160 (12)	0.0061 (9)	−0.0028 (10)
C10	0.0826 (15)	0.0570 (15)	0.0478 (10)	−0.0208 (12)	−0.0024 (10)	0.0080 (9)
C11	0.0728 (13)	0.0397 (12)	0.0450 (9)	−0.0074 (9)	−0.0084 (9)	0.0026 (8)
C12	0.0551 (10)	0.0391 (11)	0.0348 (7)	−0.0054 (8)	−0.0106 (7)	−0.0035 (7)
C13	0.0491 (10)	0.0381 (10)	0.0366 (7)	−0.0010 (8)	−0.0114 (7)	−0.0010 (7)
C14	0.0420 (9)	0.0404 (11)	0.0389 (8)	−0.0030 (7)	−0.0104 (7)	0.0011 (7)
C15	0.0537 (11)	0.0463 (13)	0.0482 (9)	0.0039 (9)	−0.0120 (8)	0.0002 (8)
C16	0.0608 (14)	0.0734 (19)	0.1053 (19)	0.0158 (13)	0.0051 (14)	−0.0091 (15)
C17	0.084 (2)	0.087 (2)	0.144 (3)	0.0024 (17)	0.0415 (19)	−0.005 (2)
C18	0.0438 (9)	0.0490 (12)	0.0485 (9)	−0.0053 (8)	−0.0103 (7)	0.0048 (8)
C19	0.0529 (11)	0.0520 (13)	0.0562 (10)	−0.0001 (10)	0.0086 (9)	0.0086 (9)
C20	0.0858 (17)	0.0825 (18)	0.0532 (11)	−0.0016 (14)	0.0033 (11)	0.0150 (12)
N1	0.0468 (8)	0.0363 (9)	0.0459 (7)	−0.0002 (6)	−0.0071 (6)	0.0036 (6)
O1	0.0561 (8)	0.0387 (9)	0.0933 (11)	−0.0070 (6)	−0.0113 (8)	0.0163 (7)
O2	0.0780 (10)	0.0467 (9)	0.0743 (9)	0.0083 (7)	−0.0106 (8)	−0.0226 (7)
O3	0.0919 (13)	0.0588 (12)	0.1065 (14)	0.0256 (10)	0.0135 (11)	0.0258 (10)
O4	0.0605 (9)	0.0577 (10)	0.0741 (9)	0.0132 (7)	0.0094 (7)	0.0034 (8)
O5	0.0545 (8)	0.0515 (8)	0.0445 (6)	−0.0091 (6)	−0.0059 (6)	0.0084 (6)
O6	0.0865 (12)	0.1080 (15)	0.0792 (11)	−0.0482 (12)	0.0073 (10)	0.0148 (11)
S1	0.0519 (3)	0.0306 (3)	0.0617 (3)	0.0003 (2)	−0.0106 (2)	−0.0024 (2)

### Geometric parameters (Å, °)

**Table:**

C1—C6	1.380 (3)	C13—C15	1.473 (3)
C1—C2	1.384 (3)	C14—N1	1.406 (2)
C1—S1	1.7492 (18)	C14—C18	1.487 (3)
C2—C3	1.375 (3)	C15—O3	1.190 (3)
C2—H2	0.9300	C15—O4	1.320 (2)
C3—C4	1.376 (3)	C16—O4	1.444 (3)
C3—H3	0.9300	C16—C17	1.445 (4)
C4—C5	1.367 (3)	C16—H16A	0.9700
C4—H4	0.9300	C16—H16B	0.9700
C5—C6	1.378 (3)	C17—H17A	0.9600
C5—H5	0.9300	C17—H17B	0.9600
C6—H6	0.9300	C17—H17C	0.9600
C7—C8	1.383 (3)	C18—O5	1.451 (2)
C7—C12	1.390 (3)	C18—H18A	0.9700
C7—N1	1.411 (2)	C18—H18B	0.9700
C8—C9	1.380 (3)	C19—O6	1.190 (3)
C8—H8	0.9300	C19—O5	1.331 (2)
C9—C10	1.374 (4)	C19—C20	1.492 (3)
C9—H9	0.9300	C20—H20A	0.9600
C10—C11	1.367 (3)	C20—H20B	0.9600
C10—H10	0.9300	C20—H20C	0.9600
C11—C12	1.396 (3)	N1—S1	1.6838 (16)
C11—H11	0.9300	O1—S1	1.4166 (15)
C12—C13	1.440 (3)	O2—S1	1.4136 (16)
C13—C14	1.366 (3)		
			
C6—C1—C2	121.34 (17)	O3—C15—O4	123.1 (2)
C6—C1—S1	119.21 (14)	O3—C15—C13	123.3 (2)
C2—C1—S1	119.39 (14)	O4—C15—C13	113.56 (17)
C3—C2—C1	118.77 (18)	O4—C16—C17	108.4 (2)
C3—C2—H2	120.6	O4—C16—H16A	110.0
C1—C2—H2	120.6	C17—C16—H16A	110.0
C2—C3—C4	120.01 (19)	O4—C16—H16B	110.0
C2—C3—H3	120.0	C17—C16—H16B	110.0
C4—C3—H3	120.0	H16A—C16—H16B	108.4
C5—C4—C3	120.91 (19)	C16—C17—H17A	109.5
C5—C4—H4	119.5	C16—C17—H17B	109.5
C3—C4—H4	119.5	H17A—C17—H17B	109.5
C4—C5—C6	120.04 (19)	C16—C17—H17C	109.5
C4—C5—H5	120.0	H17A—C17—H17C	109.5
C6—C5—H5	120.0	H17B—C17—H17C	109.5
C5—C6—C1	118.91 (18)	O5—C18—C14	107.23 (14)
C5—C6—H6	120.5	O5—C18—H18A	110.3
C1—C6—H6	120.5	C14—C18—H18A	110.3
C8—C7—C12	122.42 (18)	O5—C18—H18B	110.3
C8—C7—N1	130.14 (18)	C14—C18—H18B	110.3
C12—C7—N1	107.44 (16)	H18A—C18—H18B	108.5
C9—C8—C7	116.6 (2)	O6—C19—O5	122.7 (2)
C9—C8—H8	121.7	O6—C19—C20	126.1 (2)
C7—C8—H8	121.7	O5—C19—C20	111.21 (19)
C10—C9—C8	121.9 (2)	C19—C20—H20A	109.5
C10—C9—H9	119.1	C19—C20—H20B	109.5
C8—C9—H9	119.1	H20A—C20—H20B	109.5
C11—C10—C9	121.5 (2)	C19—C20—H20C	109.5
C11—C10—H10	119.3	H20A—C20—H20C	109.5
C9—C10—H10	119.3	H20B—C20—H20C	109.5
C10—C11—C12	118.2 (2)	C14—N1—C7	108.65 (15)
C10—C11—H11	120.9	C14—N1—S1	127.94 (12)
C12—C11—H11	120.9	C7—N1—S1	120.40 (13)
C7—C12—C11	119.40 (18)	C15—O4—C16	116.42 (19)
C7—C12—C13	107.40 (16)	C19—O5—C18	115.88 (15)
C11—C12—C13	133.20 (19)	O2—S1—O1	120.74 (10)
C14—C13—C12	108.42 (16)	O2—S1—N1	106.44 (9)
C14—C13—C15	129.06 (18)	O1—S1—N1	106.73 (8)
C12—C13—C15	122.52 (17)	O2—S1—C1	108.47 (9)
C13—C14—N1	108.09 (16)	O1—S1—C1	109.60 (9)
C13—C14—C18	129.45 (17)	N1—S1—C1	103.45 (8)
N1—C14—C18	122.22 (16)		
			
C6—C1—C2—C3	0.1 (3)	C12—C13—C15—O4	−177.13 (16)
S1—C1—C2—C3	177.33 (15)	C13—C14—C18—O5	−97.2 (2)
C1—C2—C3—C4	−1.1 (3)	N1—C14—C18—O5	89.02 (19)
C2—C3—C4—C5	1.5 (3)	C13—C14—N1—C7	0.88 (18)
C3—C4—C5—C6	−0.7 (3)	C18—C14—N1—C7	175.80 (14)
C4—C5—C6—C1	−0.4 (3)	C13—C14—N1—S1	160.98 (12)
C2—C1—C6—C5	0.7 (3)	C18—C14—N1—S1	−24.1 (2)
S1—C1—C6—C5	−176.58 (15)	C8—C7—N1—C14	179.48 (17)
C12—C7—C8—C9	0.1 (3)	C12—C7—N1—C14	−0.79 (18)
N1—C7—C8—C9	179.75 (17)	C8—C7—N1—S1	17.6 (2)
C7—C8—C9—C10	−0.5 (3)	C12—C7—N1—S1	−162.66 (12)
C8—C9—C10—C11	0.2 (3)	O3—C15—O4—C16	2.7 (3)
C9—C10—C11—C12	0.4 (3)	C13—C15—O4—C16	−177.34 (18)
C8—C7—C12—C11	0.6 (3)	C17—C16—O4—C15	160.9 (2)
N1—C7—C12—C11	−179.20 (14)	O6—C19—O5—C18	−3.3 (3)
C8—C7—C12—C13	−179.84 (16)	C20—C19—O5—C18	176.40 (18)
N1—C7—C12—C13	0.40 (18)	C14—C18—O5—C19	−170.56 (16)
C10—C11—C12—C7	−0.8 (3)	C14—N1—S1—O2	150.20 (15)
C10—C11—C12—C13	179.76 (18)	C7—N1—S1—O2	−51.75 (15)
C7—C12—C13—C14	0.13 (18)	C14—N1—S1—O1	20.02 (18)
C11—C12—C13—C14	179.66 (18)	C7—N1—S1—O1	178.06 (13)
C7—C12—C13—C15	−179.64 (15)	C14—N1—S1—C1	−95.58 (16)
C11—C12—C13—C15	−0.1 (3)	C7—N1—S1—C1	62.47 (14)
C12—C13—C14—N1	−0.62 (18)	C6—C1—S1—O2	−4.02 (18)
C15—C13—C14—N1	179.14 (16)	C2—C1—S1—O2	178.69 (15)
C12—C13—C14—C18	−175.05 (16)	C6—C1—S1—O1	129.69 (16)
C15—C13—C14—C18	4.7 (3)	C2—C1—S1—O1	−47.59 (17)
C14—C13—C15—O3	−176.9 (2)	C6—C1—S1—N1	−116.77 (15)
C12—C13—C15—O3	2.8 (3)	C2—C1—S1—N1	65.94 (16)
C14—C13—C15—O4	3.1 (3)		

### Hydrogen-bond geometry (Å, °)

**Table:**

*D*—H···*A*	*D*—H	H···*A*	*D*···*A*	*D*—H···*A*
C2—H2···O5	0.93	2.57	3.446 (2)	157
C6—H6···O2	0.93	2.50	2.875 (3)	105
C8—H8···O2	0.93	2.42	2.993 (3)	120
C11—H11···O3	0.93	2.48	3.003 (3)	116
C18—H18A···O4	0.97	2.31	2.886 (3)	117
C18—H18B···O1	0.97	2.30	2.793 (3)	111
